# Supplementary material for: GABAergic modulation of beta power enhances motor adaptation in frontotemporal lobar degeneration
Source: Alzheimers Dement. 2025 Feb 19;21(5):e14531. doi: 10.1002/alz.14531 (PMC7617437; doi:10.1002/alz.14531)
Supplement: Supplementary file 1 — Supporting Information [file ALZ-21-e14531-s001.docx]

Supplementary Material


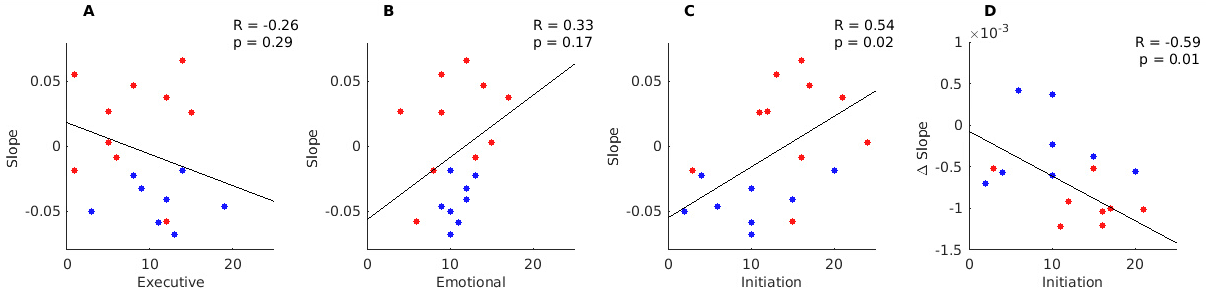


**Figure S1** **Scatter plots of the relationship in the two patient groups between task performance and Dimensional Apathy Scale scores**. A-C Plots the slope of learning on placebo, against the scores for the three parts of the DAS: executive, emotional, and cognitive and behavioural initiation aspects of apathy. D plots the change in slope of learning when on tiagabine. A positive slope is an increase in errors, and a negative slope shows a decrease in errors over trials. For all plots, PSP is plotted in red, bvFTD is in blue.


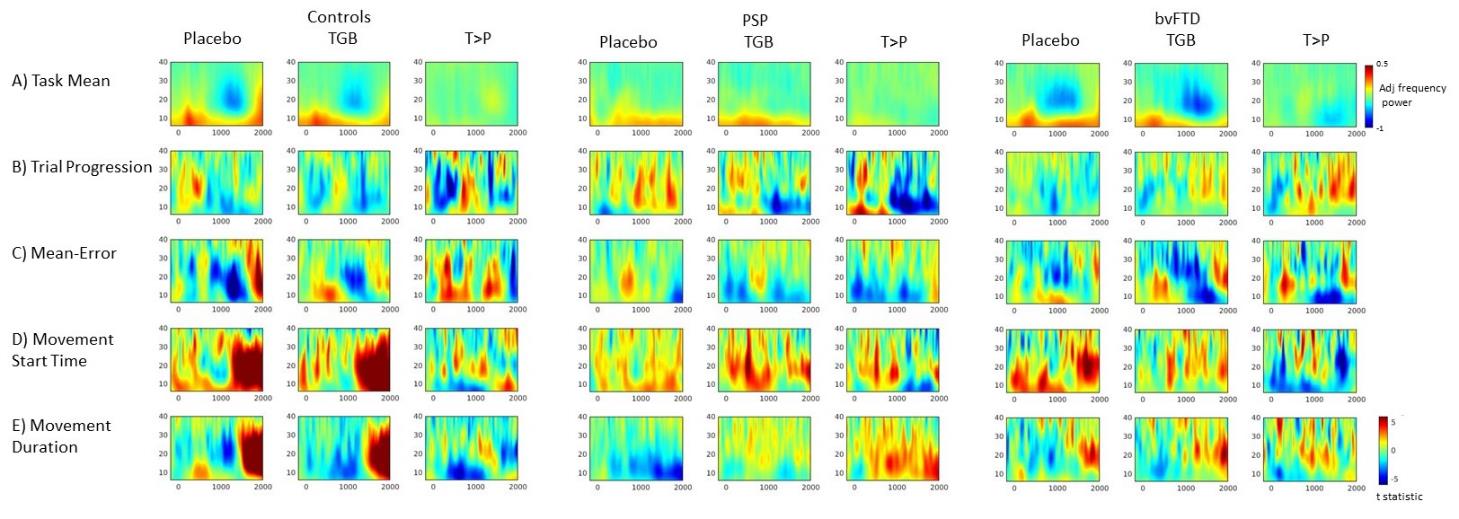


**Figure S2. Time Frequency plots showing task related changes in frequency power**. Plots show mean power for each group on placebo and tiagaine (TGB), and the difference between the two sessions. A) Group means of the Beta images of the ANOVA (adjusted frequency power) over all trials. B) Changes in power with trial progression: Red shows increases in power as trials progress, and blue shows decreases in power with trial progression. C) Mean–error: increases (red) and decreases (blue) are associated with better performance or lower mean-error. D & E show beta power increases (red) and decreases (blue) with earlier first movement, and shorter movement durations, respectively. Plots B-E are unthresholded t statistical maps.


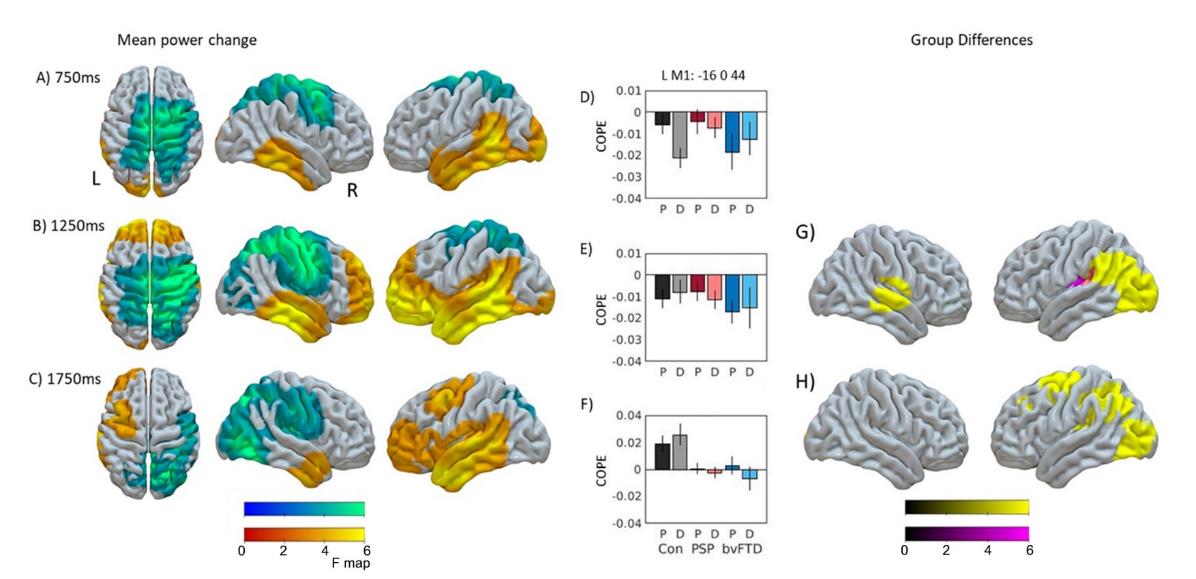


**Figure S3**. **LCMV beamformer source reconstruction of trial modulated beta power (baseline corrected).** Plots show mean source power of three time windows, centred on 750, 1250, 1750 ms. Clusters for all figures shown are familywise error corrected (*P* < 0.05 after *P* < 0.001 voxelwise uncorrected threshold). Mean power change over all groups is shown in Figures A-C. Suppression of beta power relative to baseline is shown in blue-green over bilateral pre and postentral gyrus, preSMA, and right parietal cortex. Increases in beta power relative to baseline are shown in red-yellow over anterior frontal and temporal regions. D-F) Bar Plots show peaks of cluster within left precentral gyrus (-16 0 44) for each time window. X axis is for each group on placebo (P) and Tiagabine (D), Y axis is contrast of parameter estimate, COPE. Error bars are standard error. Figures G and H show between group differences (Controls vs patients), including clusters in left motor cortex, temporal and occipital cortex (yellow) and Group x Drug interactions (violet) in the left insula, in the 1250 and 1750ms time window.


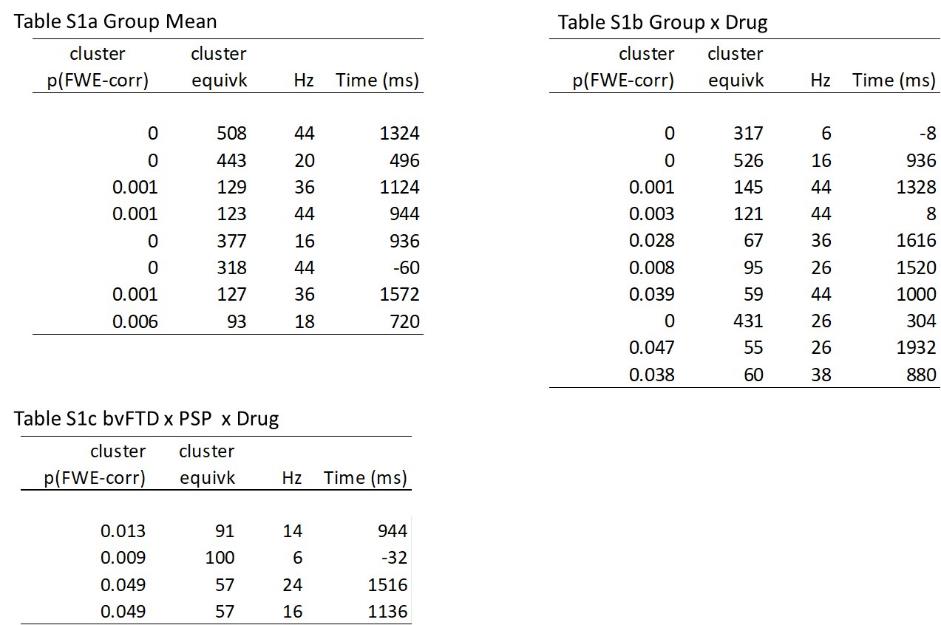


**Table S1**.Significant clusters within the time-Frequency plots in figure 3 for Trial progress, (p < 0.05 fwe cluster wise correction after p<0.001 voxel-wise threshold).


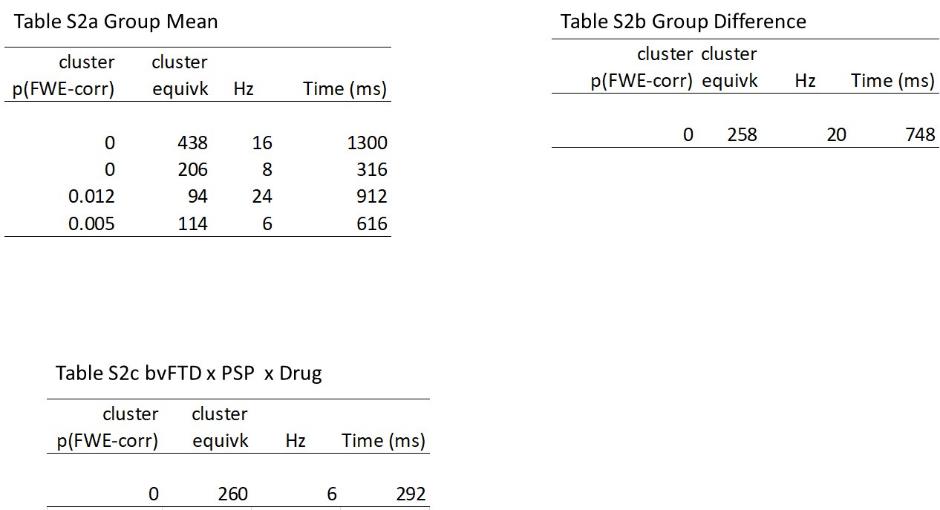


**Table S2**. Significant clusters within the time-Frequency plots in figure 3, for Mean Error (p < 0.05 fwe cluster wise correction after p<0.001 voxel-wise threshold).


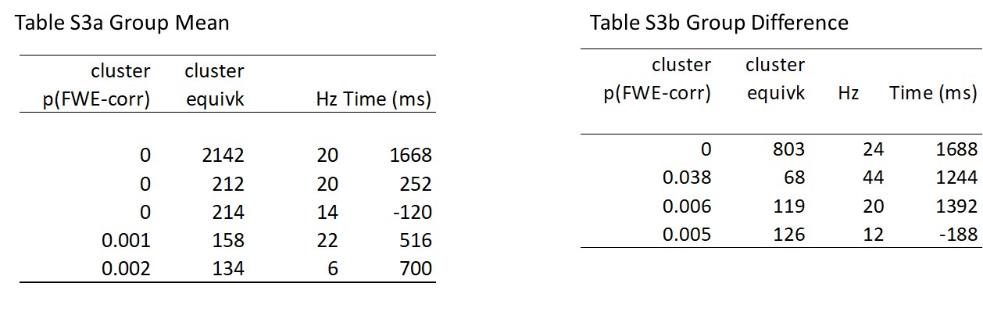


**Table S3**. Peaks of significant clusters within the time-Frequency plots in figure 3 for Movement Start Time (p < 0.05 fwe cluster wise correction after p<0.001 voxel-wise threshold).


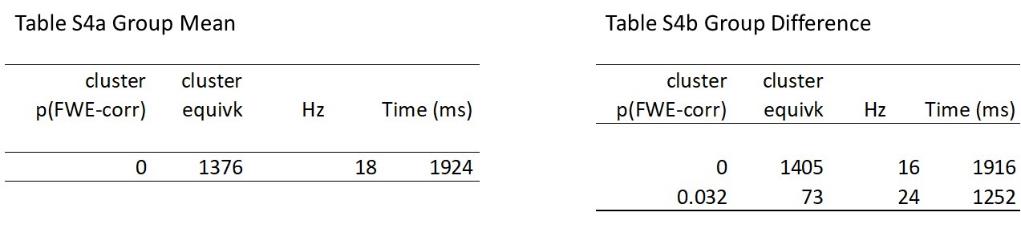


**Table S4**. Peaks of significant clusters within the time-Frequency plots in figure 3 for Movement Duration (p < 0.05 fwe cluster wise correction after p<0.001 voxel-wise threshold).
