# Supplementary material for: GABAergic modulation of beta power enhances motor adaptation in frontotemporal lobar degeneration
Source: Alzheimers Dement. Author manuscript; Available in PMC 2025 Feb 26. (PMC7617437; doi:10.1002/alz.14531)
Supplement: Supplementary file 1 [file EMS202064-supplement-Supplementary_Materials.pdf]

Supplementary Material

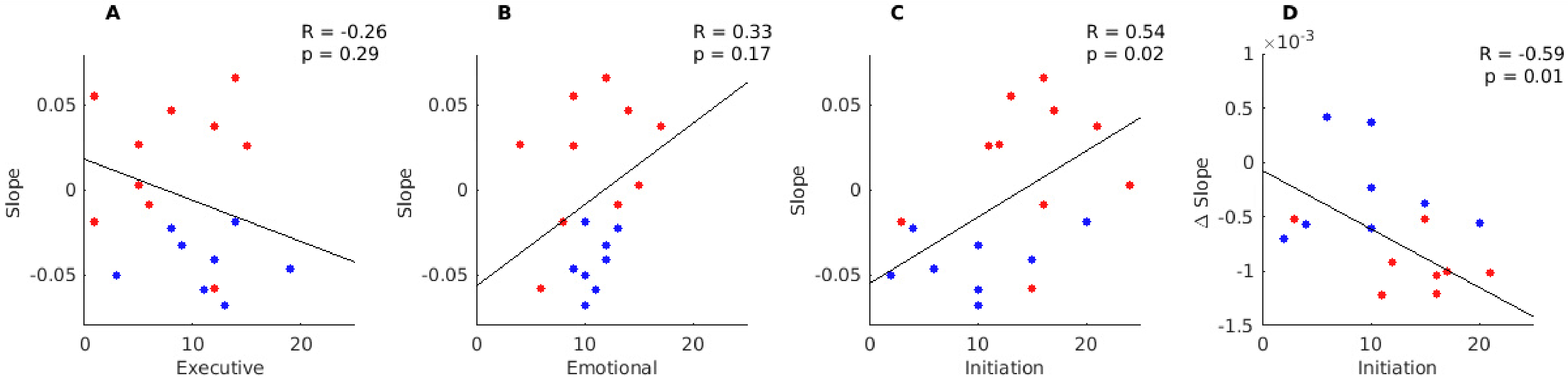

Figure S1 Scatter plots of the relationship in the two patient groups between task performance and Dimensional Apathy Scale scores. A-C Plots the slope of learning on placebo, against the scores for the three parts of the DAS: executive, emotional, and cognitive and behavioural initiation aspects of apathy. D plots the change in slope of learning when on tiagabine. A positive slope is an increase in errors, and a negative slope shows a decrease in errors over trials. For all plots, PSP is plotted in red, bvFTD is in blue.

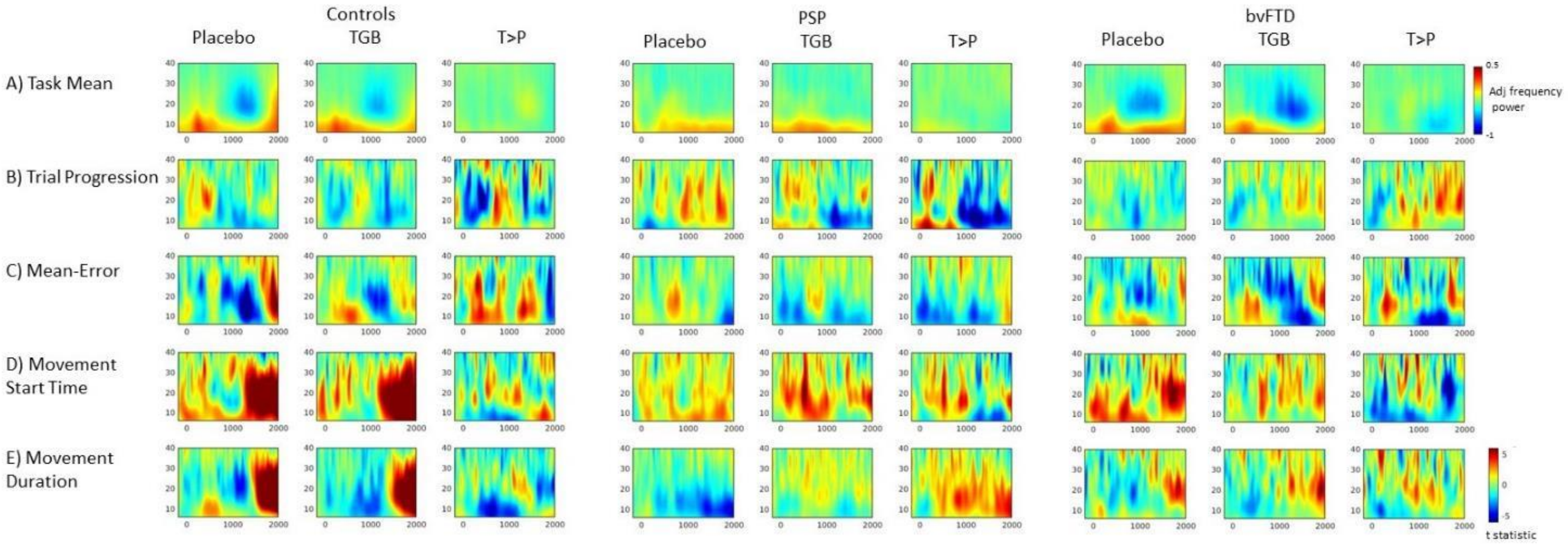

Figure S2. Time Frequency plots showing task related changes in frequency power. Plots show mean power for each group on placebo and tiagaine (TGB), and the difference between the two sessions. A) Group means of the Beta images of the ANOVA (adjusted frequency power) over all trials. B) Changes in power with trial progression: Red shows increases in power as trials progress, and blue shows decreases in power with trial progression. C) Mean–error: increases (red) and decreases (blue) are associated with better performance or lower mean-error. D & E show beta power increases (red) and decreases (blue) with earlier first movement, and shorter movement durations, respectively. Plots B-E are unthresholded t statistical maps.

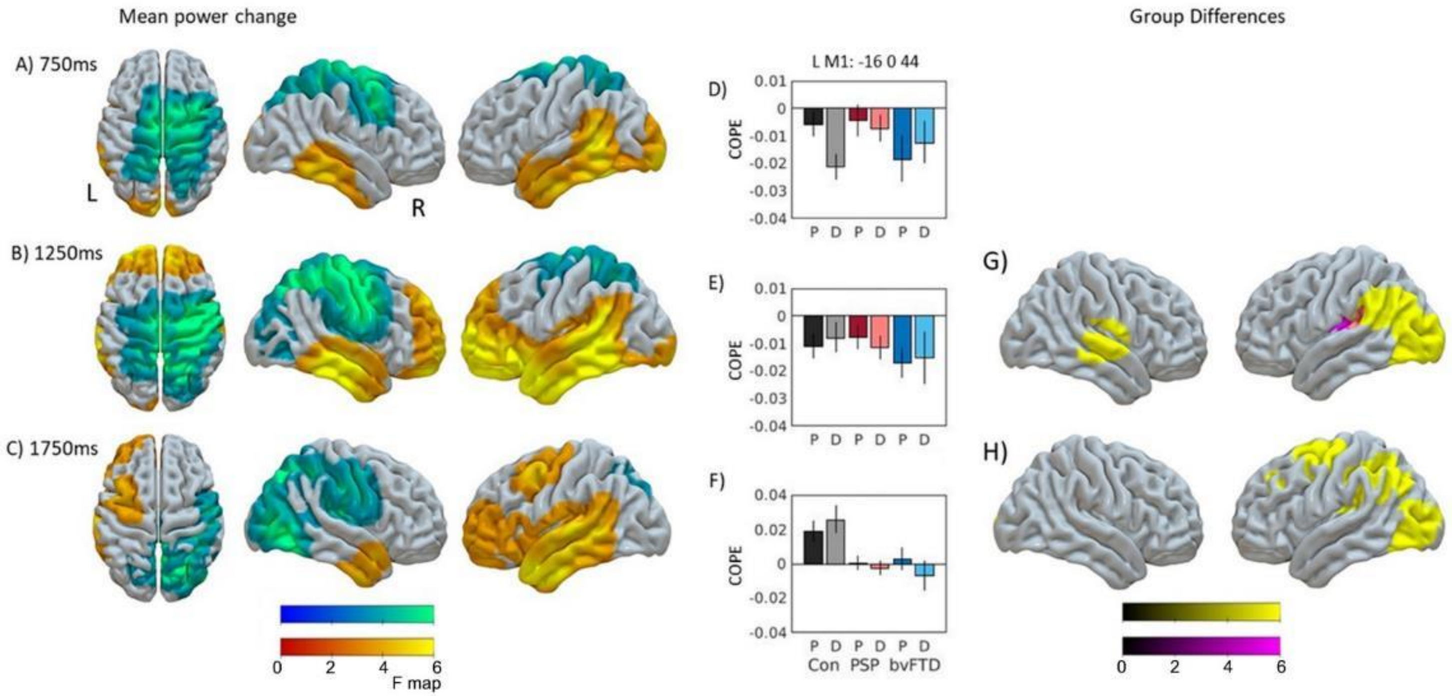

Figure S3. **LCMV beamformer source reconstruction of trial modulated beta power (baseline corrected).** Plots show mean source power of three time windows, centred on 750, 1250, 1750 ms. Clusters for all figures shown are familywise error corrected ($P < 0.05$ after $P < 0.001$ voxelwise uncorrected threshold). Mean power change over all groups is shown in Figures A-C. Suppression of beta power relative to baseline is shown in blue-green over bilateral pre and postentral gyrus, preSMA, and right parietal cortex. Increases in beta power relative to baseline are shown in red-yellow over anterior frontal and temporal regions. D-F) Bar Plots show peaks of cluster within left precentral gyrus (-16 0 44) for each time window. X axis is for each group on placebo (P) and Tiagabine (D), Y axis is contrast of parameter estimate, COPE. Error bars are standard error. Figures G and H show between group differences (Controls vs patients), including clusters in left motor cortex, temporal and occipital cortex (yellow) and Group x Drug interactions (violet) in the left insula, in the 1250 and 1750ms time window.

Table S1a Group Mean

cluster p(FWE-corr)	cluster equivk	Hz	Time (ms)
0	508	44	1324
0	443	20	496
0.001	129	36	1124
0.001	123	44	944
0	377	16	936
0	318	44	-60
0.001	127	36	1572
0.006	93	18	720

Table S1b Group x Drug

cluster p(FWE-corr)	cluster equivk	Hz	Time (ms)
0	317	6	-8
0	526	16	936
0.001	145	44	1328
0.003	121	44	8
0.028	67	36	1616
0.008	95	26	1520
0.039	59	44	1000
0	431	26	304
0.047	55	26	1932
0.038	60	38	880

Table S1c bvFTD x PSP x Drug

cluster p(FWE-corr)	cluster equivk	Hz	Time (ms)
0.013	91	14	944
0.009	100	6	-32
0.049	57	24	1516
0.049	57	16	1136

Table S1.Significant clusters within the time-Frequency plots in figure 3 for Trial progress, ($p < 0.05$ fwe cluster wise correction after $p<0.001$ voxel-wise threshold).

Table S2a Group Mean

cluster p(FWE-corr)	cluster equivk	Hz	Time (ms)
0	438	16	1300
0	206	8	316
0.012	94	24	912
0.005	114	6	616

Table S2b Group Difference

cluster p(FWE-corr)	cluster equivk	Hz	Time (ms)
0	258	20	748

Table S2c bvFTD x PSP x Drug

cluster p(FWE-corr)	cluster equivk	Hz	Time (ms)
0	260	6	292

Table S2. Significant clusters within the time-Frequency plots in figure 3, for Mean Error ($p < 0.05$ fwe cluster wise correction after $p<0.001$ voxel-wise threshold).

Table S3a Group Mean

cluster p(FWE-corr)	cluster equivk	Hz	Time (ms)
0	2142	20	1668
0	212	20	252
0	214	14	-120
0.001	158	22	516
0.002	134	6	700

Table S3b Group Difference

cluster p(FWE-corr)	cluster equivk	Hz	Time (ms)
0	803	24	1688
0.038	68	44	1244
0.006	119	20	1392
0.005	126	12	-188

Table S3. Peaks of significant clusters within the time-Frequency plots in figure 3 for Movement Start Time ($p < 0.05$ fwe cluster wise correction after $p<0.001$ voxel-wise threshold).

Table S4a Group Mean

cluster p(FWE-corr)	cluster equivk	Hz	Time (ms)
0	1376	18	1924

Table S4b Group Difference

cluster p(FWE-corr)	cluster equivk	Hz	Time (ms)
0	1405	16	1916
0.032	73	24	1252

Table S4. Peaks of significant clusters within the time-Frequency plots in figure 3 for Movement Duration ($p < 0.05$ fwe cluster wise correction after $p<0.001$ voxel-wise threshold).
